# Advanced therapy medicinal products and health technology assessment principles and practices for value-based and sustainable healthcare

**DOI:** 10.1007/s10198-018-1007-x

**Published:** 2018-09-18

**Authors:** Bengt Jönsson, Grace Hampson, Jonathan Michaels, Adrian Towse, J.-Matthias Graf von der Schulenburg, Olivier Wong

**Affiliations:** 1 Department of Economics, Stockholm School of Economics, Stockholm, Sweden; 2 Office of Health Economics, London, UK; 3 University of Sheffield, Sheffield, UK; 4 Office of Health Economics, London, UK; 5 Leibniz University Hannover, Hannover, Germany; 6 Medi-Qualite Omega, Paris, France

**Keywords:** Advanced therapy medicinal products, Regenerative medicine, Cell therapy, Gene therapy, Health technology assessment, Value

## Abstract

**Background:**

Advanced therapy medicinal products (ATMPs) are beginning to reach European markets, and questions are being asked about their value for patients and how healthcare systems should pay for them.

**Objectives:**

To identify and discuss potential challenges of ATMPs in view of current health technology assessment (HTA) methodology—specifically economic evaluation methods—in Europe as it relates to ATMPs, and to suggest potential solutions to these challenges.

**Methods:**

An Expert Panel reviewed current HTA principles and practices in relation to the specific characteristics of ATMPs.

**Results:**

Three key topics were identified and prioritised for discussion—uncertainty, discounting, and health outcomes and value. The panel discussed that evidence challenges linked to increased uncertainty may be mitigated by collection of follow-on data, use of value of information analysis, and/or outcomes-based contracts. For discount rates, an international, multi-disciplinary forum should be established to consider the economic, social and ethical implications of the choice of rate. Finally, consideration of the feasibility of assessing the value of ATMPs beyond health gain may also be key for decision-making.

**Conclusions:**

ATMPs face a challenge in demonstrating their value within current HTA frameworks. Consideration of current HTA principles and practices with regards to the specific characteristics of ATMPs and continued dialogue will be key to ensuring appropriate market access.

**Classification code:**

I.

## Introduction

The European Medicines Agency’s (EMA) definition of advanced therapy medicinal products (ATMPs) is: “medicines for human use that are based on genes or cells … [and] offer groundbreaking new opportunities for the treatment of disease and injury” [1]. ATMPs are classified into four groups: (1) gene therapy medicines; (2) somatic-cell therapy medicines; (3) tissue-engineered medicines; and (4) combined ATMPs [1]. In general, ATMPs use “… methods to replace or regenerate human cells, tissues or organs to restore or establish normal function” [2]. Mason and Dunhill comment that regenerative medicines have the potential to be “disruptive technologies” [2], and it is widely agreed that regenerative medicine is a multidisciplinary field of medicine with the potential to improve outcomes and cause a step change in the delivery of healthcare.

Health technology assessment (HTA) is a “multidisciplinary activity that systematically examines the technical performance, safety, clinical efficacy and effectiveness, cost, cost-effectiveness, organisational implications, social consequences, legal and ethical considerations” [3] of a health technology. It is a form of policy research with specific methodology that examines the short- and long-term consequences of the application of a healthcare technology to inform policy decision-making and enable rational decisions to be made for healthcare resource allocation [4]. Methodological guidelines have been developed in an attempt to harmonise the HTA process. Drummond et al. proposed a set of 15 principles that can be used in assessing existing or establishing new HTA activities [5]. These principles describe and discuss elements of good practice in developing the structure and remit of HTA organizations, the methods of and processes for conducting HTA, and the use of HTA in decision-making [5]. In addition, the European Network of HTA (EuNetHTA) has developed a value framework to enable transparent structures, procedures and standards for handling evidence and information across various forms of HTAs, economic evaluations, and other forms of assessments of the value of interventions across institutions and countries [6]. However, there is still much variation between HTA agencies [7–9].

It is anticipated that ATMPs will face some challenges in demonstrating effectiveness, cost-effectiveness and value within the HTA process. Identified challenges are associated with: clinical evidence generation, safety concerns, assessing and paying for value, uncertainty, affordability, and the manufacturing and organisation of service delivery [10–13].

ATMPs offer the potential of a one-time cure. Both comparative effectiveness data and cost-effectiveness data vs. standard of care (often long-term symptom management) will be essential to show benefit with payers increasingly averse to adding coverage for innovative technologies within limited budgets [13, 14]. Recognising the large number of ATMPs under development, HTA agencies have started to consider whether current HTA models are appropriate for the evaluation of these potentially curative therapies.

In the UK, a House of Lords Science and Technology Committee inquiry into regenerative medicine identified barriers to translation and commercialisation and recommended solutions [15]. In response to its findings the National Institute for Health and Care Excellence (NICE) commissioned a mock technology appraisal to assess whether changes to its methods and processes were needed to evaluate regenerative medicines [11]. The NICE report concluded that: (1) the existing appraisal methods and decision framework were applicable to regenerative medicines; (2) quantification of decision uncertainty was key in decision-making; (3) where uncertainty is substantial, innovative payment mechanisms may play an important role and facilitate timely patient access; and (4) choice of discount rate is extremely important and can have an impact on the incremental cost-effectiveness ratio (ICER) [11]. In a commentary on the mock appraisal of CAR T-cell therapy, Marsden and Towse challenged that while it may be possible to assess ATMPs using the existing framework, it does not necessarily follow that the framework is the most suitable means of assessment [12]. They note that ATMPs are likely to meet problems at the extremes, such as when there is substantial decision uncertainty, or when substantial clinical benefits (or cures) are offered at very high initial cost and benefits that accrue over a long-term period [12].

The objectives of this paper are to identify potential challenges that ATMPs are likely to face within current HTA methodological principles and practices in Europe and to discuss options for handling these challenges. The approach takes a broad view of HTA methodology, including the assessment of cost-effectiveness and individual and social value, as well as issues related to implementation and pricing and payment models for ATMPs in different healthcare systems.

## Methods

An Expert Panel was convened comprising members with national and international experience in research on HTA methodology and application in the UK, France, Germany, and Sweden. The Panel reviewed and identfied potential challenges in the application of principles and practices of HTA to ATMPs.

The challenges identified related to: uncertainty, discount rate, societal value vs. expected incremental health benefit, budget impact, real-world evidence, wider societal and ethical impact, and safety. Three specific areas from this list were prioritised for discussion in this paper. Prioritisation was based on the existence of methodological uncertainties that are likely to be particularly relevant to the evaluation of ATMPs.

1. *Uncertainty* Although uncertainty in the evidence is an aspect of all HTA processes, the specific nature of the evidence that is available for newly approved ATMPs is considered likely to require special consideration.
2. *Discounting* The nature of the distribution of costs and benefits was felt likely to make the estimation of cost-effectiveness particularly sensitive to decisions about appropriate discount rates.
3. *Health outcomes and value* The way in which potentially curative treatments may be considered differently to treatments that create smaller incremental benefits for larger populations may raise questions regarding the existing methods of assigning value in HTA.

The pros and cons of the alternative options in the assessment of outcome and value of ATMPs to outline potential ways forward.

### Uncertainty and advanced therapy medicinal products

#### The challenge

Cost-effectiveness analysis combines evidence on the natural course of the disease, the clinical effectiveness of alternative regimens, preferences regarding health outcomes, and the costs associated with interventions [16]. There are a number of sources of uncertainty within the available evidence that are relevant to estimating the cost-effectiveness of a particular intervention; for example, uncertainty in the treatment effects or cost inputs, the type of model used, and the applicability or generalizability of these results to a particular decision-maker [17].

In cost-effectiveness modelling sources of uncertainty include: parameter, methodological and structural [18]. Parameter uncertainty relates to the fact that the true value of a given parameter is not known. Estimates are typically based on population means of sampled data; e.g., the cost of being in a given health state, the quality of life associated with the state, the rate of clinical events over time, and relative effectiveness. In this regard, uncertainty is reflected in the standard error, confidence interval, or other representation of the probability distribution. Methodological uncertainty arises from differences in the choice of analytic methods that underpin an economic evaluation [18]; e.g., the perspective of the evaluation; handling of missing data and crossover; and, discount rates [19]. Structural uncertainty includes the judgments that have to be made when constructing and interpreting a model; e.g., the assumptions required in extrapolating costs over time [17].

Within HTA, decisions about the use of a healthcare technology are often based on the expected incremental effects and costs. Assessing the implications of decision uncertainty is an essential part of any decision-making process to provide correct evaluation of expected effect and cost; to consider whether existing evidence is sufficient; and, to assess the possible consequences of an uncertain decision for the decision-maker. Exploration of uncertainty can be carried out using: scenario analysis (e.g., different comparators, data sources, and methods), sensitivity analysis (deterministic and probabilistic), cost-effectiveness acceptability curves (CEACs; probability of cost-effectiveness), and value of information [VOI (or expected value of perfect information, EVPI)] analysis [20]. The latter looks at the benefit of collecting more evidence to assess whether additional evidence would reduce the uncertainty and guide the planning and conduct of future studies [21].

While it is possible to ask for more studies to reduce uncertainty, such studies are costly and take time. Requirements for more information and evidence before patients can get access to new treatment options must thus be evaluated according to the incremental costs and benefits of such studies. When it comes to treatments for serious and fatal diseases, the loss of patient benefits from potentially effective treatments due to delayed access must be weighed against the potential loss of benefits from the use of resources for ineffective or harmful treatments [22].

There are a number of obstacles for legislators and regulators to find the right balance between authorising early access and waiting for more trial data and delaying access [23]. Peltzman developed the theory of demand and supply, and applied it to pharmaceutical drug regulation in the US, focusing on the costs of making mistakes of two types: (1) admitting unsafe drugs, and (2) delaying the access to effective drugs [24]. While the US pharmaceutical market at that time was mainly private out-of-pocket payment, the theory applies in a similar way to today’s market with dominantly public and private third-party payment. One difference is that costs dominate over side-effects in the definition of the public interest. The majority of ATMPs are developed for well-defined groups of patients with severe illnesses, and the same patient will experience both the positive and negative consequences. The resource allocation decision is about comparing and valuing uncertain outcomes from new treatment for some patients vs. uncertain outcomes for other patients. This is a difficult but unavoidable choice, and decisions about pricing and reimbursement determines the incentives for (what type of) innovation. When new drugs are developed for specific groups of patients with severe disease with limited available treatment options, based on understanding of disease mechanisms and what may work, the pressure for early access is understandable.

While uncertainty is unavoidable when allocating resources for health, as with most other investment decisions in the private and public sector, there are ways to manage this uncertainty to provide suitable incentives for both buyer and seller. Regulatory decisions about market authorization have developed since the 1970s to include follow-up studies and re-assessment. For public and private third-party payments, there is the additional opportunity to make contracts that include actions based on generating or analysing information about relevant outcomes. Such new contractual arrangements are of particular importance for providing rational incentives for development and use of ATMP. However, the shift to outcome-based contracts is not necessarily a simple step. Insights from contract theory, rewarded by the Nobel prize in Economics in 2016 [25], tell us that great care needs to be taken when introducing outcome-based contracts. The key to success is careful planning and collection of relevant and unbiased data.

Traditionally, drug development has assumed that all patients with a particular condition respond similarly to a given drug. While there often is evidence that not all patients respond in the same way, there are often limited possibilities for determining response in advance. Personalised medicine, however, recognises that complex diseases should no longer be considered as a single entity and also provides instruments to differentiate using, for example, molecular diagnostics [26]. One disease may have many different forms, or ‘subtypes’ which will not only vary between patients who have the same disease but also within an individual patient as they get older and their body changes. For the understanding of value for new drugs with a potential for cure, there is also the option to use information on deep response for modeling the overall survival (OS) benefit.

Different ATMPs are likely to face different evidence generation challenges. For some, clinical effectiveness evidence will necessarily come from small, single-arm, or single-centre early phase clinical trials. Populations with rare, severe or advanced disease may be very small, and therefore, adequate recruitment to larger scale clinical trials would take a lot of time at great expense. In addition, some ATMPs are being tested in a wide range of differing indications each with small numbers, and, for some, safety and efficacy evidence may read across from earlier approvals. For others, identifying an appropriate comparator may be difficult, particularly where no alternative treatments are available, or where alternative treatments are not available and treatment is for a life-threatening condition, and randomisation to a control group may be unethical [12, 27]. Reliance on evidence from single-arm trials or other observational evidence may, therefore, be required. Non-randomised evidence is generally less well received by HTA agencies due to the higher risk of bias and higher uncertainty surrounding effect estimate sizes [11]. However, marketing authorisations have already been granted to ATMPs without evidence from randomised controlled trials (e.g., chimeric antigen receptor T-cell therapy [CAR-T]), and for orphan drugs little difference has been observed in the approval rate based on the level of evidence presented [28].

Trials evaluating ATMPs may also be more dependent on surrogate outcomes rather than clinical outcomes [29]. Surrogate endpoint data; e.g., progression-free survival, are quicker and easier to acquire than final clinical endpoint data; e.g., overall survival, enabling shorted trials and quicker regulatory review. However, there is often considerable uncertainty because they may not capture the combined benefit–risk profile of a technology and a surrogate may not translate to benefits for a clinical endpoint. It is also important to validate surrogate endpoints in both licensing and coverage or reimbursement decisions by establishing the level of evidence, assessing the strength of the association, and quantify the relationship between surrogate and final outcomes [30].

The implications of the problems associated with the availability of limited evidence will largely depend on both the level of unmet need in the studied population and the likeliness of cure or improvement without experimental treatment [11]. Marketing authorisations may be based on limited evidence of clinical effectiveness, requiring decision-makers to extrapolate benefits over the longer term.

#### Potential solutions

Several authors have observed the problems for HTA agencies for predicting long-term survival for ATMSs and pointed to the need for developing new methods that more accurately capture the value of new innovative drugs that might include treatment to cure for some fraction of the treated patients [31, 32]. For new treatment to cure innovations, the traditionally used parametric methods will underestimate survival, primarily when a plateau of long-term survival is observed, and therefore, give misleading estimates of life expectancy [33].Othus et al. propose a method where survival is measured separate for cured and non-cured patients [34]. Applying the method to ipilimumab, reduced the cost per QALY estimate to about a third [35].

For ATMPs, the collection of real-world evidence via registries—disease or treatment—will be essential for the management of uncertainty in outcomes and value. Post-marketing surveillance is usually a safety requirement accompanying regulatory approval [36]. Collection of evidence post-launch will be key to demonstrating evidence of effectiveness and comparative effectiveness over the longer term. Other complementary evidence that could be used includes natural history data, utility data, and the use of pooled data. Despite the resistance of HTA agencies to accepting non-randomised comparative evidence, there are several flexibilities within current HTA framework in respect of acceptable levels of evidence. For example, in England, the National Institute for Health and Care Excellence (NICE) framework allows for the consideration of non-randomised evidence where it is difficult to conduct randomised controlled trials in the assessment of highly specialised technologies [37]. In Sweden, the new system for managed entry includes a protocol for follow-up for all drugs accepted within this access scheme. Such data are used for market access agreements and as information for renewed decisions [38].

Identified methodological uncertainties are amenable to empirical research. While additional research may suggest methodological changes to resolve structural uncertainties that are particularly relevant to ATMPs, a potential disadvantage is that this might require changes to current HTA processes and raise concerns about comparability with previous appraisals.

Uncertainty could also be addressed in the decisions taken. In a recent publication, Hampson et al. recommend the consideration of outcome-based agreements and their possible combination with potential methods of leased payments when health benefits are expected over a long time horizon [10]. Performance-based risk-sharing arrangements (PBRSAs) are sometimes used where there will be significant budget impact and where there is uncertainty in the available evidence base [39–41]. PBRSAs include outcome-based schemes, risk-sharing agreements, coverage with evidence development, access with evidence development, conditional licensing and managed entry schemes. However, structuring a PBRSA is not straightforward; for example, where patients have co-morbidities and measurement of benefit may be more complex than a straightforward yes or no [40].

Amortisation or leasing schemes whereby upfront payment systems are replaced with a series of payments spread over the expected duration of benefit from a given health technology, subject to the health technology delivering the anticipated benefit, offer one approach [42]. Gottlieb and Carino argue that leasing mechanisms help to align the cost with the long-term benefits allowing the treatment to be funded while balancing their budgets and note that such arrangements are commonplace with medical equipment where cost is spread over the time horizon that the equipment will be used [42]. Although this approach could potentially be beneficial there is still a requirement to pay for an uncertain benefit [42]. One way around this would be to combine annualisation with a risk-sharing agreement [13]. The potential drawbacks must be considered alongside the benefits of amortization models [13].

### Discounting in the evaluation of the cost-effectiveness of ATMPs

#### Rationale for discounting

In economic evaluation costs and outcomes of two or more healthcare technologies are compared over time. It is, therefore, important that the impact of differential timing of costs and benefits is accounted for when the decision is made [43]. Discounting formalizes the adjustment of future values to current value; it accounts for the differential timing of costs and benefits by weighting them according to when they are accrued.

In most cases, it is a standard practice to apply the same discount rate to costs and benefits (uniform), and to keep the discount rate constant over time. Most studies apply discount rates provided by central institutions, such as HTA agencies, departments of finance or mutually accepted guidelines for evaluation studies. However, the approach is widely debated: should future cost and benefits should be discounted at the same rate, should discount rates be the same in the assessment of acute care or preventive care, should discounting be done at all? Uniform discounting is supported by two main arguments: consistency thesis (which proposes that inconsistencies may occur when discounting at two different rates) [44], and the postponement paradox, whereby Keeler and Cretin argue that if health benefits are discounted at a lower rate than costs, the cost-effectiveness ratio can be improved by delaying the introduction of the technology in question and continue to be improved by further delays [45]. The alternative is differential discounting whereby health benefits are discounted at a typically lower rate than costs, and variable discounting whereby the rate is altered over time.

#### Discount rates in practice

Although the discount rate is a crucial parameter in many policy decisions, it is often assigned with little explicit justification [14]. Almost all HTA bodies in Europe employ discounting in HTA applying an annual discount rate of between 3% and 5% for costs and between 1.5 and 5% for health benefits [46]. A mix of uniform and differential discount rates are used: seven HTA agencies specify uniform discounting for costs and benefits [47–53], while three HTA agencies require higher discount rates for costs than health benefits [54–56]. As a general rule, agencies require the discount rate to be varied in sensitivity analyses to examine the sensitivity of the results to the discount rate. In Sweden, recommendations suggest that sensitivity analysis should include use of discount rates of 0% and 5%, as well as a calculation where costs are discounted by 3% and health effects by 0% [52]. In England, there is some flexibility in the NICE process when “… treatment restores people who would otherwise die or have a very severely impaired life to full or near full health, and when this is sustained over a very long period (normally at least 30 years) … a non-reference-case discount rate for costs and outcomes may be considered”. A discount rate of 1.5% for costs and benefits may be considered by the Appraisal Committee if it is highly likely that, on the basis of the evidence presented, the long-term health benefits are likely to be achieved” [50].

Whether the discount rate should be the same in all evaluation studies on public projects in a country or whether it should be different for, say infrastructure projects, health services, and medical care programs is an open question. In addition, whether the discount rate used in evaluation studies should correlate with the general level of interest rates on financial markets is another question. The latter implies that in times of high interest rates, the cost–benefit ratio of preventive care programs would look worse than in times of low interest rates.

ATMPs are likely to involve high intervention costs occurring years before all the health effects have emerged. Due to the temporal distribution of costs and benefits, the choice of discount rate has a potentially significant effect on estimates of cost-effectiveness. Should specific discounting rules therefore be applied for ATMPs?

To answer this question, one should keep in mind the three main arguments for discounting future costs and benefits with a positive discount rate: (1) opportunity cost of capital, (2) time preference of individuals or public decision-makers, and (3) decreasing marginal utility of income [57]. The first, opportunity cost, comes from investment theory. If a budget is spent for Project A, it cannot be spent for Project B. If Project B leads to a rate of return of, say x %, Project A should have at least the same return rate. Interest rates on capital markets show these opportunity costs of capital, or more generally the benefit of an alternative given up—or benefit forgone—when a decision is made. The second, time preference, is an individual’s willingness to prefer a certain amount of money or a certain utility now than the same amount of money or utility in the future [57]. In general, society prefers to benefit sooner rather than later, preferring to offset the risk that something will prevent or reduce the chance of future consumption. For example, 1 day in perfect health today may be considered better than 2 days in the distant future [57]. The third, decreasing marginal utility, reflects positive economic growth, or the decrease in the relative value of a good/service over time [57]. If wealth and income increases over time, future generations will be richer than current ones, so a gain today is better for society than the same gain tomorrow. Time preference and opportunity cost underlie attempts to define a “social discount rate”: the rate at which future costs and benefits of publicly funded programmes should be discounted [57].

It is not necessarily the case that the arguments for employing a positive discount rate for decisions at the individual patient or investor level justify discounting in projects where society as a whole bears the costs and gains the benefits. If ATMPs are evaluated from an individual point of view, individual time preferences should be used. If ATMPs are evaluated from a societal point of view, because they are financed by a public healthcare or health insurance scheme, the societal time preference should be used instead. Individual time preference can be elicited using stated preference methods. They are likely to be situation specific, and thus to vary widely. Individuals are also mortal and, as such, it is not surprising that individuals prefer present to future consumption. Society on the other hand is not mortal and thus the societal time preference is perhaps more appropriate when considering the sustainable use of resources for current and future generations. The social rate of time preference also relates to preferences of society as a whole for present over future consumption. Thus, typically the discount rate for healthcare programs is based on a social time preference.

This argument has already been developed in a theoretical framework by Arrow and Lind in the Arrow-Lind Theorem [58]. According to this theorem, the social cost of the risk moves to zero under certain assumptions as the population tends to infinity, so that projects can be evaluated on the basis of expected net benefit alone [58]. The three assumptions of the Arrow–Lind Theorem are: (1) the government covers all costs initially.; (2) the return of the project must be independent of individual income; and, (3) the benefits must be spread out over a reasonably large number of individuals [58]. All three assumptions hold true in a society where healthcare costs are financed by the government or social health insurers [58].

As discussed in the early economic literature by Baumol and Tullock, the discount rate should be low or zero in the evaluation of projects with high externalities [59, 60]. This might apply for treatments with high future gains for the society and vaccination programs [59, 60], and is why one might argue that the discount rate should be zero or lower than individual’s time preferences in evaluations of long-term public projects or programs.

A lower discount rate for health benefits should be considered whereby benefits are discounted at a lower rate than costs. It is argued that discounting health benefits at a lower rate than costs takes into account any potential increase in the future value of health effects [61]. Based on assumptions about the pure utility discount rate, the elasticity of marginal utility, the growth rate of income, and the extent to which health affects income, it has been estimated that the discount rate on health effects should be 1–3.5% lower than the discount rate on costs [61]. Claxton *et al*. argue that the choice of discount rate depends on a number of factors: on whether the social objective is to maximise discounted health outcomes or the present consumption value of health; on whether the budget for healthcare is fixed; on the expected growth in the cost-effectiveness threshold; and on the expected growth in the consumption value of health [62]. They demonstrate that if the budget for healthcare is fixed and decisions are based on incremental cost effectiveness ratios (ICERs), discounting costs and health gains at the same rate is correct only if the threshold remains constant. Expecting growth in the consumption value of health does not itself justify differential rates but implies a lower rate for both.

A lower discount rate (in comparison to a higher one) will support health programmes or technologies with costs now and outcomes far in the future, and will also favour the benefits of future generations.

### Does “value” need to be considered differently for ATMPs?

#### Assessing value using the QALY

Priority setting in healthcare has long been recognized as: “an intrinsically complex and value-laden process” [63]. Society, including relevant stakeholders, such as patients, providers, insurers, and citizens, has a wide range of social values and interests that result in different perceptions of what makes health interventions valuable [64]. A recent review has also identified heterogeneity in value assessment systems across Europe, which results in significant differences in coverage recommendations across settings based on how HTA agencies perceive or interpret evidence and the associated uncertainties [65]. Current HTA value assessment frameworks do not adequately capture the range and diversity of stakeholder values. Frameworks used by HTA agencies are typically based on comparative clinical benefit assessment and economic evaluation (cost utility analysis or cost-effectiveness analysis) as the main method of determining the value of new technologies. Sometimes, this involves reflecting health gain as quality-adjusted life years (QALY), or an alternative patient-relevant outcome; for example, life-years gained [65].

Typically, individuals with ill health want improved health; i.e., length of life and/or quality of life (patient experience in respect of, for example, delivery of care, pain or other factors, impact on the family, time off work). QALYs combine quality and quantity of life into a single composite measure: value per health state multiplied by the length of time in that state [66]. QALYs can be used to compare the benefits of interventions across therapy areas and determine priorities by means of cost per QALY ratios [66] against a threshold value of a QALY. In theory, this approach ensures that new treatments do not displace more health gain than they provide and do not diminish the overall value of benefits gained from the healthcare budget. Thresholds are in place in England, Poland and an academic proposal for a threshold was identified for Spain in a recent review [65]. In England, the threshold ranges between £20,000 to £30,000 [50] although evidence indicates that this threshold can be modified in practice with some products with an ICER below £20,000 receiving a negative recommendation and other products with an ICER above £30,000 receiving a positive recommendation [65].

While the threshold provides a framework for decision-making, it does not take into account the fact that decision-makers may wish to account for other factors not captured in the cost-effectiveness ratio when assessing a medicine pricing/reimbursement decision [67]. One way of managing this has been to vary the threshold ICER according to the type of medicine, the type of disease, and the decision-making context, such as the age of the patient or the severity of the disease [37]. However, what constitutes value varies between jurisdictions. For example, in England, NICE uses an end of life criterion for which the underlying assumption is that society places a higher priority on treating those patients near the end of their life [50]: an additional QALY under end of life conditions is worth more than a QALY at another point time although this is widely debated [68]. The threshold is also higher for “ultra-orphan” health technologies [69].

In Norway, patient benefit, resource use and severity of the disease are proposed as the three main sources of value for priorities in the healthcare system [70]. Rarity is not mentioned as a specific criteria for value. In Sweden, society should be able to pay for more health gain and accept lower standards for scientific evidence when prioritising drugs for treating rare conditions if the following conditions are met: (1) the treatment has a high cost per health gain as a consequence only treating a few patients; (2) it involves a health condition with a very high level of severity; (3) that the treatment option being considered is assumed, based on firm grounds, to have a substantial effect, and (4) that no alternative treatment having a substantial effect is available [71]. The Norwegian and Swedish proposals have broad public and political support, but should only been seen as general guidelines. It is up to Legemiddelsverket in Norway and TLV in Sweden to translate these guidelines into specific decisions for individual products applying for reimbursement. That includes making the trade-off between different dimensions of value.

Guidelines for reimbursement are silent about the role of empirical studies for assessing the value of specific products or classes of products from patients or the general public. Such studies did not find consistent support for a preference for health gains to cancer patients in the context of health maximization [72, 73]. However, they observed a higer willingness to pay for severe diseases, while the empirical support for additional value for “end-of-life” treatments are weak [74].

There is also evidence that with short life expectancy, few will trade off less survival vs. improved quality of life [75]. Cost per life year-gained may, therefore, be a more adequate metric for assessing the value of new treatments for persons with short life expectancy. Presenting a calculation of cost per LYG in addition to cost per QALY, would provide decision-makers with additional information for decisions of value from a patient perspective.

#### Additional elements of value (beyond health gain)

While assessments using the cost per QALY approach include many important and relevant aspects of value such as impact on survival, quality of life, and potential cost savings, they do not include assessment of other elements of value beyond the health gain for the patient and costs to the health system which may also be relevant for payers, patients, and society [76]. Examples of these wider benefits include disease severity, age of onset, lifetime burden of illness, socioeconomic impact, and possible spillovers from the initial innovation [65], or improvements in the quality of or process of care that may also not be captured by measures of improvements in outcome (e.g., home vs. hospital treatment or oral vs. intravenous treatment). Some ATMPs may not be deemed cost-effective according to conventional decision rules, and in some cases this will be because the therapy simply does not represent good value for money. However, in other cases, the therapy may offer high value that is just not reflected in the HTA evaluation, and/or the therapy may be restricted by the set of aforementioned challenges (e.g., difficulties in establishing robust estimates of clinical effectiveness). Potential additional sources of value are explored in the following paragraphs, each of which could potentially justify some premium on top of what is deemed acceptable to pay for pure health gains.

Short life expectancy at diagnosis or at the start of treatment is a common feature of many illnesses. In healthcare, a tension sometimes arises between the injunction to do as much good as possible with scarce resources (“cost-effectiveness”) and the injunction to rescue identifiable individuals at immediate risk, regardless of cost (the “Rule of Rescue”) [77]. Culyer notes that the “rule of rescue” argument has two important caveats: first, the strength of the argument is weakened if the additional time is of poor quality or of a worse quality than would be the case under normal palliative care, and, second, that the end-of-life argument applies to all patients, and not only cancer patients, in which context it is commonly discussed [76].

In an end of life context, patients themselves may also be willing to take risks or pay for options with greater uncertainty or immediate mortality risk if there is a significant chance of increased long-term survival [78]; this has been referred to as the “value of hope” [79]. Literature on prospect theory also suggests that patients with life-threatening conditions sometimes appear to make risky treatment decisions as their condition declines, contradicting the risk-averse behaviour predicted by expected utility theory [80]. Rasiel et al. demonstrated that behaviour links to a reference point; for example, when the recently diagnosed patient has not yet adapted to their new prognosis, the prospective value of the investigational therapy exceeds that of the conventional therapy [80]. Patients’ reference points may take time to adjust following a change in diagnosis, with implications for predicting under what circumstances a patient may select experimental or conventional therapies or select no treatment [80].

While prospect theory captures individual valuations when faced with difficult choices under uncertainty, the application of the theory to inform public decisions has its challenges. Suppose, for example, a diagnostic was developed that can predict which patients will be cured by a therapy. This may reduce the value from an individual patient perspective, since the hope of cure for all patients is replaced by certain cure for a few. A public payer may still be interested in paying for this test, thus reducing the cost of treatment, but there may be perverse incentives against the development of companion diagnostics that may reduce the size of the market since this takes away the “value of hope”.

Curative therapies could eliminate the need for long-term management and provide longer term increases in quality of life. It is not known, however, whether curative therapies are valued more highly by society than treatments that offer the same “total” health gains through marginal gains over many years and/or patients. Little evidence is available to suggest whether or not this preference does exist, and no weighting is currently available to adjust for this [10].

Additional elements of value identified in the literature in relation to complementary diagnostics may also be relevant for consideration in the assessment of ATMPs include: innovation and real option value, and scientific spillovers [79]. These elements are briefly discussed below; however, there is currently limited research into how these elements should be formally incorporated into the decision-making framework.

Producing innovative drugs is increasing in cost. The role of cost-effectiveness analysis in incentivizing innovation is controversial; currently cost effectiveness analysis rewards gains in clinical benefit. Innovation has been defined as the: “successful introduction of something new and useful, for example, introducing new methods, techniques, or practices or new or altered products and services” [81]. It is typically characterised in the economic literature as “a cumulative process of success” [82]. In healthcare, the term innovation lacks specificity and differs by country [83]. Italy has published criteria for identifying an innovative product. With this algorithm, pharmaceuticals are designated as an important, moderate, or modest therapeutic innovation based on: (i) the availability of existing products or (ii) the extent of the therapeutic benefit [83]. In France, an improvement of medical benefit Amerlioration du Service Medical Rendu (ASMR) level (major innovation, important imIf ATMP improvement, significant improvement, minor improvement and no improvement) is assigned for each product vs. standard of care, but the criteria used to determine these levels is not defined [84]. Innovations that result in benefits for society or facilitate benefits from future technologies might justify some reward [81]. Culyer argues that innovation is “already rewarded (or at least encouraged) through the patent system and profit with special pricing and profit regulatory scheme in most countries” [76]. To best assess the value of innovation, perhaps consideration of the overall health need alongside innovation goals and priorities is required [81].

Real option value refers to the investment in healthcare that can lead to potential treatment pathways for patients in the future as other new technologies become available [79]. Evidence suggests that patients perceive “option value” from treatment as getting one treatment despite its disadvantages (e.g., side effects) increases the likelihood of benefiting from a better treatment in the future [10, 79].

Finally, scientific spillovers relate to knowledge spillover whereby one company’s achievements can lead to the success of another company developing a similar technology [79, 85]. Sweeney and Goss suggest that the market authorisation of a new therapy leads to additional research and the generation of additional evidence to understand the benefits of a treatment in a given clinical context [79, 85]. Combinations of approved therapies are used in successive clinical trials, further increasing scientific spillovers over time [79, 85]. The advantages and disadvantages of the benefits of scientific spillovers are, however, unpredictable.

To summarise, traditional cost-effectiveness analysis conducted as part of HTA focuses on life-years gained, improvements in patient quality of life, and cost savings within healthcare. While the current framework may be appropriate for the assessment of ATMPs, it is also important that the full potential value of ATMPs is recognised. We suggest that this will involve incorporating an assessment—or at least consideration—of other aspects of value into the current evaluative framework for reimbursement. However, if such additional considerations are to be included in future assessment, then further work is required to ensure that these considerations are also applied to competing technologies and those that may potentially be displaced by new expenditure on ATMPs [86].

## Recommendations

Following analysis and discussion of the prioritised HTA methodological topics in relation to the specific characteristics of ATMPs the following recommendations have been made.

1. Consideration of outcome-based contracts, whereby collection of follow-up data can mitigate the increased uncertainty related to market access decisions for ATMPs.
2. The development of registries for ATMPs to collect long-term data regarding outcomes, adverse events, resource use and costs. Ideally these should be:
  - Independent of specific technologies and manufacturers.
  - Inclusive of all technologies, patient populations and indications.
  - International, or based upon an internationally agreed set of data definitions to allow subsequent aggregation and meta-analysis.
  - Funded through approval being given as conditional on data submission, where existing evidence is inconclusive.
3. Consideration of the use of EVPI analysis as an adjunct to HTA, to inform better decision-making in regard to approvals that are conditional on further evidence collection.
4. Development of consistent methods for conditional approval based upon ‘coverage with evidence’ or risk sharing, and clear arrangements for the collection and submission of supplementary evidence and review.
5. The establishment of an international, multi-disciplinary forum to consider the economic, social and ethical implications of the choice of differential or joint discount rates for costs and benefits for HTA, in a variety of circumstances. Such consideration may be extended to the implications of discount rates in wider contexts, such as public health, environmental and infrastructure policy decisions.
6. Review existing evidence or, where necessary, commissioning further primary research to establish preferences regarding aspects of ‘value’ that may not currently be adequately captured in calculations of QALYs. Such considerations include:
  - Valuation of ‘cure’ as opposed to wider incremental benefits.
  - Social value beyond health gain.
  - Patient preferences for treatments beyond health gain.
  - Process utilities and ‘option value’.
  - The value of spillovers linked to innovation.

Such studies would also need to consider the impact of incorporating these additional aspects of value on previous HTA decisions and in relation to the opportunity costs of displaced activities and disinvestment.

## Conclusions

ATMPs may face challenges with current HTA principles and practices. Consideration of ways of dealing with increased uncertainty; for example, by developing outcome-based payment models, and dialogue regarding the economic, social, and ethical aspects of the implications of discounting given the differential between payment of costs and receipt of benefits, will be key. In particular, ATMPs may face a challenge in demonstrating value within current evaluative frameworks. Broadening the definition of “value” to systematically capture elements of value not captured in the QALY, and the importance of considering the value of ATMPs and the value forgone in other disease areas, if resources are switched when other elements of value are taken into consideration will be key to facilitating appropriate market access.

The proposed recommendations are put forward to initiate and continue the dialogue around HTA for ATMPs in context of other published reports. By following these recommendations, the opportunity exists to improve the HTA methods used for the assessment of ATMPs which would enable healthcare systems to manage some of the uncertainties presented by early data from these products.
